# Genetic variability evaluation and cultivar identification of tetraploid annual ryegrass using SSR markers

**DOI:** 10.7717/peerj.7742

**Published:** 2019-09-20

**Authors:** Gang Nie, Ting Huang, Xiao Ma, Linkai Huang, Yan Peng, Yanhong Yan, Zhou Li, Xia Wang, Xinquan Zhang

**Affiliations:** Department of Grassland Science, College of Animal Science and Technology, Sichuan Agricultural University, Chengdu, China

**Keywords:** Annual Ryegrass, Cultivars Identification, Genetic Diversity, SSR Marker

## Abstract

Annual ryegrass (*Lolium multiflorum*) is a widely used cool-season turf and forage grass with high productivity and ornamental characteristics. However, the abundant intra-cultivar genetic variability usually hampers the application of conventional techniques for cultivar identification. The objectives of this study were to: (1) describe an efficient strategy for identification of six tetraploid annual ryegrass cultivars and (2) investigate the genetic diversity based on SSR markers. A total of 242 reliable bands were obtained from 29 SSR primer pairs with an average of 8.3 bands for each primer pair and the average value of polymorphic information content (PIC) was 0.304. The result of analysis of molecular variance (AMOVA) revealed that 81.99% of the genetic variation occurred in within-cultivars and 18.01% among-cultivars. The principal coordinate analysis (PCoA) showed that the first two principal axes explain 8.57% (PC1) and 6.05% (PC2) of total variation, respectively. By using multi-bulk strategy based on different filtering thresholds, the results suggested that bands frequency of 40% could be used as a reliable standard for cultivar identification in annual ryegrass. Under this threshold, 12 SSR primer pairs (00-04A, 02-06G, 02-08C, 03-05A, 04-05B, 10-09E, 12-01A, 13-02H, 13-12D, 14-06F, 15-01C and 17-10D) were detected for direct identification of six tetraploid annual ryegrass cultivars, which could be incorporated into conservation schemes to protect the intellectual property of breeders, ensure purity for consumers, as well as guarantee effective use of cultivars in future.

## Introduction

Annual ryegrass (*Lolium multiflorum*) is one of the most important turf and forage grasses with large areas of utilization around the world. As a cool-season annual bunchgrass with high productivity, palatability and ornamental characteristics (Vieira et al., 2004), annual ryegrass is considered to be a sustainable grass species in livestock systems, landscaping as well as ecosystem services (Castanheira et al., 2014). Like most outcrossing species, annual ryegrass cultivars are propagated by intercrossing selected plants and traits are improved through several generations of random mating in limited germplasm (Busti et al., 2004). However, the long-term intraspecific hybridization and continuous popularization of the hybrid varieties led to a narrow genetic background and inbreeding depression among the annual ryegrass cultivars. Phenotypic and genetic similarity among many cultivars severely restricted the application of traditional techniques for cultivar identification based on morphological characters. Therefore, the development of a reliable approach for annual ryegrass cultivar identification is becoming increasingly significant.

DNA-based molecular markers have provided a powerful method for genetic diversity assessment (Badr, El-Shazly & Mekki, 2012; Srinivas et al., 2012) and cultivar identification (Anand, Prabhu & Singh, 2012; Karaagac et al., 2014). Although genetic diversity of ryegrass accessions have been evaluated by amplified fragment length polymorphism (AFLP) (Guthridge et al., 2001), restricted fragment length polymorphisms (RFLP) (Sato et al., 1995), sequence-related amplified polymorphism (SRAP) (Huang et al., 2014a; Huang et al., 2014b) and randomly amplified polymorphic DNA (RAPD) (Bolaric et al., 2005; Vieira et al., 2004), simple sequence repeat (SSR) markers are still considered as a powerful tool for characterizing the genetic variability of cultivars. SSRs have been widely used in various crop and grass species, including wheat (*Triticum aestivum*), rice (*Oryza sativa*), potato (*Solanum tuberosum*), *Hemarthria altissima* and orchardgrass (*Dactylis glomerata*) due to its high polymorphism, co-dominance inheritance, and high reproducibility (Róder et.al, 2002; Anand, Prabhu & Singh, 2012; Karaagac et al., 2014; Huang et al., 2014a; Huang et al., 2014b; Xie et al., 2011a; Xie et al., 2011b; Jones et al., 2001).

Cultivar-specific DNA-based molecular markers are generally difficult to identify in outcrossing species due to their high within-cultivar genetic variability (Busti et al., 2004). Bulking strategy, which combines DNA of multiple samples within a given group, could provide a valuable tool to increase the frequency of rare alleles (Forster et al., 2001; ReyesValdes et al., 2013). In maize (*Zea mays*) populations, a comparison analysis was made between individual genotyping and the bulk approach to estimate SSR allele frequencies, and the results showed that SSR analysis of bulks was more stable and cost-effective for large-scale molecular characterization of germplasm collections (Reif et al., 2005). Also, previous studies proved that bulking strategy with molecular markers could effectively analyze the genetic diversity and identify the cultivars in tall fescue (*Festuca arundinacea*) (Tehrani et al., 2009), alfalfa (*Medicago sativa*) (Herrmann, Flajoulot & Julier, 2010), white clover (*Trifolium repens* L.) (Kölliker et al., 2001), orchardgrass (*Dactylis glomerata*) (Jiang et al., 2013) and maize (Dubreuil et al., 2006).

Nevertheless, with bulking strategies, the loci had to be filtered for suitable estimation of allele frequencies. Many previous studies on genetic diversity evaluation and cultivar identification of outcrossing plants were carried out using single bulk strategy without replications, which induced unstable results for its application (Badr, El-Shazly & Mekki, 2012; Jiang et al., 2013). Furthermore, although bulking DNA samples has been successfully applied in several plant species to distinguish cultivars and to study genetic diversity (Tehrani et al., 2009; Herrmann, Flajoulot & Julier, 2010; Dubreuil et al., 2006; Jiang et al., 2013; Kölliker et al., 2001), a comparison study is necessary to identify optimal sampling strategies and to select informative loci.

The objectives of this study were to: (1) describe an efficient strategy for identification of six tetraploid annual ryegrass cultivars in China and (2) investigate genetic variability among and within the cultivars based on SSR markers. Results from this study could provide useful information for future annual ryegrass variety protection program.

## Material and Methods

### Plant materials

The plant materials used in this study included six tetraploid major commercial cultivars of annual ryegrass which were registered in China (Table 1). Seeds of each cultivar were germinated in a petri dish (Biosharp™, Hefei, China, diameter= 90 mm) with two layers of moistened filter paper. The germination was performed in a growth chamber with a constant temperature of 25 °C. One hundred robust seedlings of each cultivar were selected to transplant into a sand-peat mixture (Panshi™, He’nan, China). All the seedlings were grown in a greenhouse with a 12 h photoperiod at average temperatures of 25/20 °C (day/night) for two weeks. Plants were watered every two days and not fertilized during the experiment.

**Table 1:** Information of six annual ryegrass varieties used in the study.

No.	Cultivars	Origins	Ploidy
1	Angus 1	DLF-Trifolium Group	Tetraploid
2	Changjiang No.2	Sichuan Agricultural University	Tetraploid
3	Abundant	DLF-Trifolium Group	Tetraploid
4	Tetragold	DLF-Trifolium Group	Tetraploid
5	Double Barrel	DLF-Trifolium Group	Tetraploid
6	Aderenalin	Seed Force CO., LTD, New Zealand	Tetraploid

### DNA extraction

For the DNA extraction, fresh young leaves were collected from each individual and a total of 10 independent bulks were constructed for each cultivar. For each bulk, samples of 10 randomly selected leaves were mixed. Genomic DNA was extracted using the genomic DNA extraction kit (Tiangen^®^, Beijing, China) according to the manufacturer’s protocol, then the quality and concentration of the DNA were measured on 0.8% (w/v) agarose gels and NanoDrop ND-1000 spectrophotometer (NanoDrop Technologies Inc., Rockland, DE, USA), respectively. DNA samples were diluted to 20 ng µl^−1^ and stored at 4 °C for PCR amplification.

### SSR amplification

A total of 29 pairs of SSR primers selected from Hirata et al. (2006) were used in this study (Table 2, Supplemental Information 1). PCR amplifications were carried out in a volume of 15 µl containing 1.5 µl (20 ng µl^−1^) genomic DNA, 7.5 µl PCR-Mixture (10 × reaction buffer, 2.0 mM Mg^2+^, 0.6 mM of each dNTP, Tiangen^®^, Beijing, China), 1.2 µl (10 pmol µl^−1^) forward and reverse primer mixture, 0.3 µl of Taq DNA polymerase, and 4.5 µl of ddH_2_O. PCR amplification reaction was performed using touchdown mode with the following program: 5 min at 94 °C for 1 cycle, followed by 10 cycles at 94 °C for 30 s, varying annealing for 45 s according to annealing temperature (Tm) of different primers (Table 2) with each cycle decreased by 0.5 °C, and 72 °C for 1 min, and then 30 cycles at 94 °C for 30 s, annealing temperature for 45 s, and 72 °C for 1min, final extension at 72 °C for 7 min, and then stored at 4 °C. The amplified fragments were separated on 6% denatured polyacrylamide gel electrophoresis using 200V pre-electrophoresis for 30 min, and then 400V electrophoresis for 2 h. After electrophoresis, the gel was stained by AgNO_3_ solution and photographed by camera.

**Table 2:** The information of the SSR primers used in this study and the results of amplification for annual ryegrass cultivars.

**Primer pairs No.**	**AT** (°C)	**PS (bp)**	**TNB**	**NPB**	**PPB (%)**	**PIC**
LMgSSR00-04A	63	219	10	10	100	0.421
LMgSSR01-01E	65	319	4	4	100	0.176
LMgSSR01-02H	65	161	9	9	100	0.344
LMgSSR01-06D	67	174	11	10	90.9	0.389
LMgSSR01-09C	65	148	10	9	90.0	0.320
LMgSSR01-10G	65	353	5	5	100	0.317
LMgSSR02-05G	65	197	8	5	62.5	0.279
LMgSSR02-06G	65	234	8	8	100	0.371
LMgSSR02-07D	65	187	6	6	100	0.240
LMgSSR02-08C	63	161	12	12	100	0.373
LMgSSR03-04E	65	361	7	5	71.4	0.328
LMgSSR03-05A	65	273	13	13	100	0.390
LMgSSR04-05B	67	195	14	14	100	0.270
LMgSSR04-09D	63	167	10	9	90	0.230
LMgSSR07-01D	56	401	5	5	100	0.278
LMgSSR07-07G	65	219	8	8	100	0.308
LMgSSR09-09C	65	180	7	4	57.1	0.190
LMgSSR09-10H	65	337	4	4	100	0.235
LMgSSR10-09E	65	205	9	7	77.8	0.334
LMgSSR12-01A	65	356	7	7	100	0.361
LMgSSR13-02H	65	244	9	9	100	0.380
LMgSSR13-07A	65	152	11	10	90.9	0.172
LMgSSR13-12D	65	270	8	8	100	0.270
LMgSSR14-06F	65	266	9	8	88.9	0.403
LMgSSR15-01C	63	223	10	10	100	0.386
LMgSSR16-01E	65	236	3	3	100	0.267
LMgSSR16-06G	65	241	10	10	100	0.286
LMgSSR17-04E	65	179	9	9	100	0.170
LMgSSR17-10D	65	247	6	6	100	0.333
Total	–	–	242	227	–	–
Average	–	–	8.3	7.8	93.8	0.304

**Notes.**

AT Annealing temperature PS Predicted size TNB Total number of bands NPB Number of polymorphic bands PPB Percentage of polymorphic bands PIC Polymorphic information content

### Data analysis

The amplified fragments of all SSR primers were scored as band presence (1) or absence (0) to form binary matrix, and each of them was treated as an independent character regardless of its intensity. The total number of bands (TNB), number of polymorphic bands (NPB), and percentage of polymorphic bands (PPB) were calculated. The discriminatory power was evaluated by polymorphic information content (PIC) calculated as ${\mathrm{PIC}}_{i}=2{f}_{i} \left( 1-{f}_{i} \right) $, where PIC_*i*_ is the polymorphic information content of marker ‘*i*’, *f*_*i*_ is the frequency of the amplified allele (band present), and 1 − *f*_*i*_ is the frequency of the null allele (Roldan–Ruiz et al., 2000).

The analysis of molecular variance (AMOVA) was used to estimate the genetic variation within and among cultivars in WIN AMOVA software (Excoffier, Smouse & Quattro, 1992). The genetic similarities among 10 bulks within each cultivar were calculated with NTSYS-pc2.10 software. The principal coordinate analysis (PCoA) and the un-weighted pair group method with arithmetic mean (UPGMA) dendrogram were constructed by NTSYS-pc 2.10 software and MEGA v6.0 (Tamura et al., 2013), respectively.

In order to define the optimal threshold of bands frequencies for ryegrass cultivar identification, the comparison analysis of difference thresholds for filtering statistical bands was conducted. The bands appeared 10 times (100% frequencies), nine times (90% frequencies), eight times (80% frequencies), seven times (70% frequencies), six times (60% frequencies), five times (50% frequencies), four times (40% frequencies), three times (30% frequencies), two times (20% frequencies) and one time (10% frequencies) within 10 bulks of each cultivar and were respectively recorded to form a new data matrix. The Dice’s similarity coefficients for estimating genetic similarities between cultivars were determined by using NTSYS-pc 2.10 software, and the UPGMA dendrogram were constructed for each filtering statistical data matrix. Each pair of primers was integrated with a single fingerprint pattern for evaluating the discriminatory power.

## Results

### Genetic variation and clustering analysis of the six tetraploid annual ryegrass cultivars

**Figure 1:**
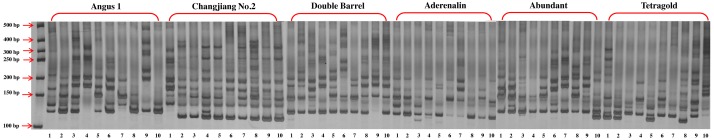
The gel electrophoresis picture of primer LMgSSR00-04A for PCR amplification among 10 bulks for six annual ryegrass cultivars.

A total of 242 reliable bands were obtained from 29 SSR primer pairs, with an average of 8.3 bands for each primer ranging from 3 (LMgSSR16-01E) to 14 (LMgSSR04-05B) (Fig. 1, Table 2). Among them, 227 bands (93.8%) were polymorphic and the percentage of polymorphic bands (PPB) per primer pair ranged from 57.1% to 100%. Meanwhile, the polymorphic information content (PIC) values for each primer ranged from 0.170 (LMgSSR17-04E) to 0.421 (LMgSSR00-04A), with an average of 0.304, demonstrating potentially sufficient discriminatory capacity. The AMOVA analysis was conducted to investigate the genetic variation within and among cultivars. The results revealed that the majority of the total variation was due to within-cultivars (81.99%), while variance among cultivars was only responsible for 18.01% (Table 3).

**Table 3:** Analysis of molecular variance (AMOVA) of the cultivars based on original data.

Source of variation	Degree of freedom	Sum of square	Mean square deviation	Variance component	Percentage of total variance	*P*-value
Among cultivar	5	516.49	103.30	7.10	18.01%	<0.001
Within cultivar	54	1744.71	32.31	32.31	81.99%	<0.001
Total	59	2261.19	135.61	39.41	100%	

**Figure 2:**
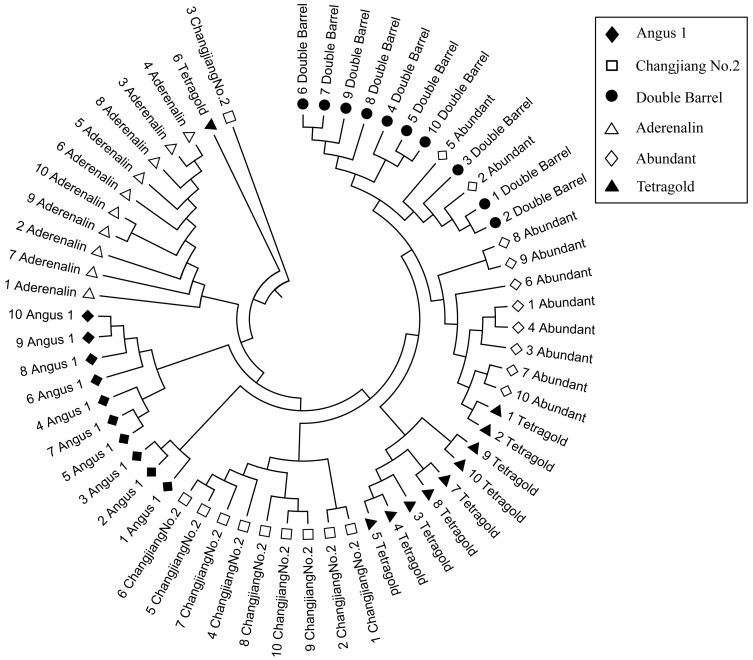
The Unweighted Pair Group Method with Arithmetic Mean (UPGMA) dendrogram of each bulks for six annual ryegrass cultivars based on GS data.

The genetic similarity (GS) analysis among 10 bulks of each cultivar showed that the variation within cultivars was different and the GS coefficient value ranged from 0.639 (relative high variation within a cultivar) for the cultivar ‘Angus 1’ to 0.723 (relative low variation within cultivar) for the cultivar ‘Aderenalin’ (Supplemental Information 2). The Un-weighted Pair Group Method with Arithmetic Mean (UPGMA) dendrogram based on GS data showed that all six cultivars with 10 bulks were obviously distinguishable (Fig. 2). Among them, the cultivars ‘Double Barrel’ and ‘Abundant’ had closest family relationship with two bulks of ‘Abundant’ clustered with ‘Double Barrel’ group, followed by ‘Tetragold’, ‘Changjiang No.2’ and ‘Angus 1’. The cultivar ‘Aderenalin’ was divided from the other cultivars, indicating that the highest genetic distance and different parental ancestry compared to others. One bulk of ‘Changjiang No.2’ and one bulk of ‘Tetragold’ separated from other bulks of their respective cultivars to form a separate branch, respectively.

**Figure 3:**
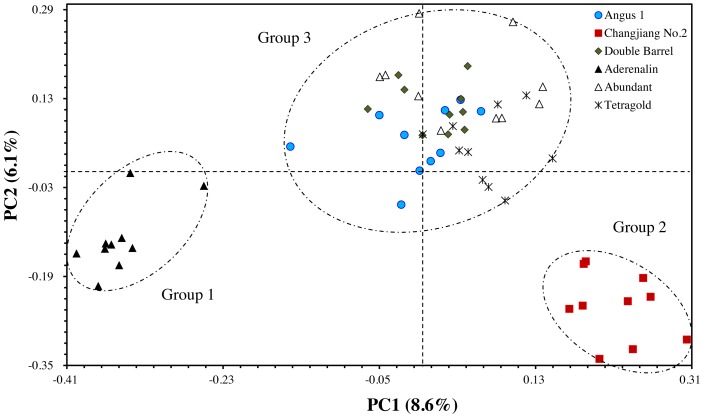
The principal coordinate analysis (PCoA) of six annual ryegrass cultivars based on GS data. The different filtering thresholds of the bands frequencies was (a) 10%, (b) 20%, (c) 30%, (d) 40%, (e) 50%, (f) 60%, (g) 70%, (h) 80%, (i) 90% and (j) 100%.

The principal coordinate analysis (PCoA) provided better understanding of the relationships among the tested annual ryegrass cultivars. The results showed that the total variation could well be explained by the first two principal axes, with explanation rate of 8.57% (PC1) and 6.05% (PC2), respectively. The results of PCoA were consistent with the cluster analysis that the bulks of cultivar ‘Aderenalin’ were significantly distinguished by PC2 (group 1). ‘Changjiang No.2’ could be clearly separated by PC1 (group 2), while UPGMA dendrogram based on GS data did not distinguish it. The other cultivars were distributed around the origin of coordinates forming a group 3 (Fig. 3).

### Cultivar identification of tetraploid annual ryegrass

Although multi-bulk strategy could increase the frequency of rare alleles, the identification of a suitable threshold for filtering scored bands is a guarantee for stable results. In this study, the comparison analysis of difference thresholds among 10 independent bulks of each cultivar was conducted, and the results showed that the identification ability varied among SSR primer pairs and different filtering strategies (Table 4). Based on difference filtering threshold, the mean of directly distinguishable number of studied cultivars ranged from 1.4 (100% frequencies) to 4.5 (30% frequencies), and comparable identification abilities were observed when scored bands appeared two, three, or four times among 10 bulks (the number of cultivars could be directly distinguished was above four).

**Table 4:** The comparative analysis of different bulk strategy threshold of bands for the cultivar identification.

**Primer pairs No.**	**10%**	**20%**	**30%**	**40%**	**50%**	**60%**	**70%**	**80%**	**90%**	**100%**
LMgSSR00-04A	6	6	6	6	6	6	4	3	3	1
LMgSSR01-01E	0	0	2	2	1	1	1	0	1	3
LMgSSR01-02H	4	2	4	4	4	2	4	4	2	2
LMgSSR01-06D	3	6	3	4	6	6	4	6	2	1
LMgSSR01-09C	4	6	6	4	6	6	4	4	2	2
LMgSSR01-10G	4	3	2	3	3	2	1	0	1	3
LMgSSR02-05G	3	6	6	4	6	6	4	4	1	1
LMgSSR02-06G	6	6	6	6	4	6	6	3	1	1
LMgSSR02-07D	4	4	4	4	1	0	1	1	1	0
LMgSSR02-08C	6	6	6	6	6	6	4	2	2	1
LMgSSR03-04E	2	3	4	4	4	6	2	3	1	1
LMgSSR03-05A	2	6	4	6	6	3	1	1	2	2
LMgSSR04-05B	6	6	6	6	3	2	2	2	1	0
LMgSSR04-09D	6	4	4	2	1	0	0	0	0	0
LMgSSR07-01D	4	2	4	4	2	0	0	2	2	1
LMgSSR07-07G	1	4	6	4	2	2	4	4	6	4
LMgSSR09-09C	2	3	4	4	2	1	0	1	1	0
LMgSSR09-10H	1	1	0	1	0	1	2	2	2	2
LMgSSR10-09E	3	6	6	6	4	6	4	1	1	1
LMgSSR12-01A	4	6	6	6	3	1	0	0	0	0
LMgSSR13-02H	4	6	6	6	6	3	6	6	6	4
LMgSSR13-07A	6	6	6	3	1	1	0	0	0	2
LMgSSR13-12D	6	6	6	6	6	2	2	1	0	0
LMgSSR14-06F	2	4	6	6	6	6	4	3	0	0
LMgSSR15-01C	6	6	6	6	6	4	2	2	1	0
LMgSSR16-01E	0	0	1	1	0	0	0	1	1	0
LMgSSR16-06G	6	6	4	3	2	2	2	2	3	3
LMgSSR17-04E	2	2	3	3	2	1	0	0	1	6
LMgSSR17-10D	3	6	4	6	3	2	2	1	1	0
Average	3.7	4.4	4.5	4.3	3.5	2.9	2.3	2.0	1.6	1.4

Among the 29 SSR primer pairs, 9 of them could effectively identify six cultivars when the bands appeared frequency at 60%, 10 at 50%, 12 at 40%, 14 at 30% and 16 at 20%, whereas just two of them were available for directly identifying six cultivars at 70%, 80% and 90% (Table 4). In addition, the identification ability could always be improved by a combination of different primer pairs. For example, at the threshold of 40%, all six cultivars could be identified by a combination of the following primers (primer 02-05G could identify ‘Angus 1’, ‘Changjiang No.2’, ‘Aderenalin’ and ‘Tetragold’, while primer 07-07G could identify ‘Angus 1’, ‘Double Barrel’, ‘Aderenalin’ and ‘Abundant’).

### Suggestions for optimum multi-bulk strategy

**Figure 4:**
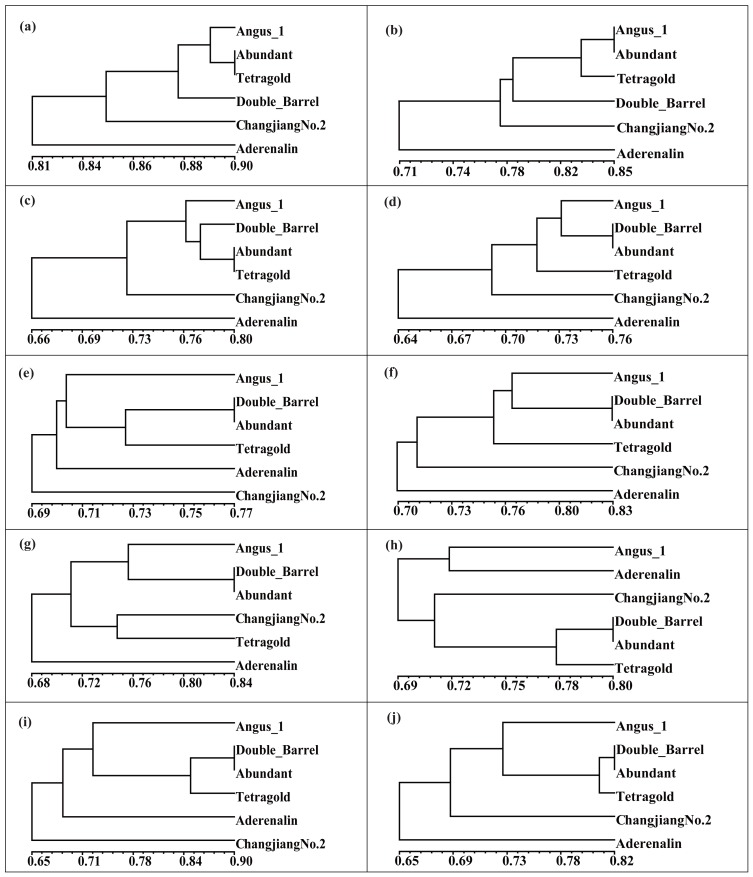
The Unweighted Pair Group Method with Arithmetic Mean (UPGMA) dendrogram of six annual ryegrass cultivars based on different filtering threshold. The different filtering threshold of the bands frequencies was (a) 10%; (b) 20%; (c) 30%; (d) 40%; (e) 50%; (f) 60%; (g) 70%; (h) 80%; (i) 90%; (j) 100%.

To validate the effectiveness of multi-bulk strategy results, clustering analysis of binary scoring with difference thresholds was conducted. The UPGMA dendrogram showed that difference filtering strategies produced varied identification ability, while more stable results could be achieved with higher band presence frequency (Fig. 4). Interestingly, the two most related cultivars were the ‘Abundant’ and ‘Tetragold’ when the bands appeared at a frequency of 10% with GS coefficient value of 0.90, ‘Abundant’ and ‘Angus 1’ at 20% with GS coefficient value of 0.85, ‘Abundant’ and ‘Tetragold’ at 30% with GS coefficient value of 0.80, and were stably ‘Abundant’ and ‘Double Barrel’ after the bands appeared frequency above 40% with GS coefficient value ranging from 0.76 to 0.90. Moreover, the results showed that ‘Aderenalin’, introduced from Germany, was the most genetically distant cultivar from others, except when DNA bands appeared at the frequency of 50% and 90%, ‘Changjiang No.2’ was the most genetically distant one. ‘Changjiang No.2’ is a cultivar hybridized between ‘Aubade’, introduced from USA, and ‘Ganxuan No.1’, bred from diploid breeding line by chromosome doubling and radiation mutation by mass selection in China.

Although those cultivars with limited available pedigree information could be difficult to assess in regard to phylogenetic relationships, the SSR markers were sufficient for distinguishing the cultivars. In most filtering situations, the UPGMA dendrogram (Fig. 4) showed that all six examined cultivars were grouped into three clusters with ‘Aderenalin’ was clearly distinguished from the others and followed by ‘Changjiang No.2’ which was consistent with previous cluster (Fig. 2) and PCoA (Fig. 3) based on original data binary.

Taking into account the ability to differentiate the cultivars (the discriminating power) and the bands stability (the consistency of DNA fingerprints), the results suggested that DNA bands presence frequency of 40% could be used as a reliable threshold when using multi-bulk strategy for cultivar identification. In this study, 12 of 29 SSR primer pairs (00-04A, 02-06G, 02-08C, 03-05A, 04-05B, 10-09E, 12-01A, 13-02H, 13-12D, 14-06F, 15-01C and 17-10D) were available for directly identifying six cultivars and ‘Abundant’ and ‘Double Barrel’, introduced from the USA had the closest genetic relationships, followed by ‘Angus 1’ introduced from the USA, ‘Tetragold’ introduced from the USA, ‘Changjiang No.2’ and ‘Aderenalin’ introduced from Germany.

## Discussion

Accurate identification of cultivars is necessary for protecting the intellectual property of breeders, ensuring purity and effective use of cultivars. However, it would be misleading to evaluate DNA fingerprints of several populations only through a diversity measure such as PIC, because hidden noise is not considered (ReyesValdes et al., 2013). Therefore, identification of cultivars with strong cross-pollination using molecular markers could not be simply conducted in a conventional manner. SSR marker is a traditional and mature technique for DNA fingerprint construction (Herrmann, Flajoulot & Julier, 2010; Laidò et al., 2013), and it has been successfully applied to genetic diversity and cultivar identification in crops (Róder et.al, 2002; Anand, Prabhu & Singh, 2012; Karaagac et al., 2014), horticultural plants (Tsai et al., 2013; Xie et al., 2011a; Xie et al., 2011b), and forages (Tehrani et al., 2009; Herrmann, Flajoulot & Julier, 2010; Jiang et al., 2013).

Although SSR markers provided an attractive approach for varietal identification through allele frequencies in diploid ryegrass (Wang et al., 2009), cultivar identification at the individual level was still difficult when using allele frequencies due to high genetic variation within cultivars. Furthermore, tetraploid genotypes were preferred over diploid genotypes in ryegrass breeding programs because of enhanced biomass production and improved forage quality resulting from chromosome doubling (Sanderson & Elwinger, 2004). In this study, 81.99% of the total variation was attributable to within-cultivar genetic variation, which showed consistent results with previous study in cross-pollinated plants. In perennial ryegrass cultivars, substantial genetic variation existed and the total within-cultivar component of genetic variation was 85.35% (Kubika et al., 2001). In tetraploid white clover, the within-cultivar variation explained 84% of the total variation, while variance among cultivars was only responsible for 16% of the total variation (Kölliker et al., 2001). In addition, although the mean PPB based on band frequencies used in this study was 75.1%, which was lower than that determined using SSR for orchardgrass cultivars (92.1%) (Jiang et al., 2013) and *Elymus breviaristatus* (83.4%) (Gu et al., 2015), but the primers still confirmed sufficient discriminatory capacity.

Bulking strategy could provide a valuable tool to deal with rare-occurring bands induced by within-cultivar variability (Forster et al., 2001; ReyesValdes et al., 2013). Optimal sample size is critical for bulking strategy when estimating genetic diversity and cultivar identification. Studies based on random amplification polymorphic DNA (RAPD) analysis indicated that the bulk size was 20 for red clover (*Trifolium pratente* L.) (Ulloa, Ortega & Campos, 2003) and 30 for perennial ryegrass (*Lolium perenne* L.) (Sweeney & Danneberger, 1994). Ghérardi et al. (1998) analyzed 40 bulked individuals to characterize populations of tetraploid alfalfa, while Jiang et al. (2013) used 25 bulked samples to distinguish populations of orchardgrass. Alleles at frequencies of more than 5% may be effectively sampled by 100 individuals (Forster et al., 2001), and the 100 plants bulk size has been proved to be suitable for cultivar identification of creeping bentgrass (*Agrostis stolonifera* L.) (Caceres et al., 2000) and tall fescue (Busti et al., 2004). In maize, the evaluation of allele frequencies from band analysis in three independent DNA bulks of 10 random individuals each, allowed diversity estimation and cultivar identification (Dubreuil et al., 2006). In summary, various sample sizes and sampling strategies have been used in previous studies and there is not yet a consensus method to define sampling schemes from preliminary data on allele frequencies.

However, the drawback of the bulking strategy is that bulking more than 10 samples will affect stable statistical results, while small bulk size increased the sampling variance, which renders an inconsistent bulk-based fingerprint due to insufficient sensitivity of the marker protocol (Forster et al., 2001; Larson, Jones & Jensen, 2004; ReyesValdes et al., 2013). van Etten et al. (2008) used 10 bulked samples to estimate the genetic diversity of maize in communities of the western highlands of Guatemala. Yu & Pauls (1993) also indicated that alfalfa cultivars were distinguished from each other on the basis of at least one unique RAPD marker with bulked DNA samples from 10 individuals, and recommended that DNA bulking of 10 individuals would be useful for identifying cultivars among heterogeneous populations. Results of this study demonstrated that SSR markers could be effectively used to estimate genetic diversity and cultivar identification of tetraploid annual ryegrass. This study suggested that bulking size of 10 plants and allele frequencies of 40% among 10 bulks could be used as reliable parameters when using multi-bulk strategy in annual ryegrass. This is consistent with the strategy suggested in annual ryegrass that allele frequencies of 40% is an optimal threshold for scoring allele to construct DNA fingerprint and cultivar identification (Nana, Maiko & Koi, 2008).

The six commercial cultivars selected in this study were popular in China and were registered with China National Committee for Approval of Grass Varieties from 2004–2012. Although they were introduced from different countries and have different genetic backgrounds, the results showed that the variation was mainly distributed within-cultivar. It seems that the six annual ryegrass cultivars population was small, and the findings from the current study may not hold for larger sample size, but the suggested strategy allowed us to evaluate general tendencies of allele frequencies and variation among and within cultivars for DNA bulked SSR marker information in this species. In previous studies, the identification of three to eight cultivars was also estimated by using molecular markers in *Hemarthria*, strawberry (*Fragaria ananassa* Dutch), perennial ryegrass, and creeping bentgrass (*Agrostis stolonifera* L.) cultivars (Huang et al., 2014a; Huang et al., 2014b; Gidoni et al., 2006; Kubika et al., 2001; Caceres et al., 2000). In addition, with multiple analyses and validation, this study provided a guideline for genetic diversity assessment and cultivar identification of highly outcrossing plants in SSR marker system, and 12 identified SSR markers for directly distinguishing six annual ryegrass cultivars could be incorporated into conservation schemes and further studies involving a large number of cultivars are yet to be tested.

## Conclusions

In this study, a total of 242 reliable bands were obtained from 29 SSR primer pairs to estimate genetic variation, and results showed that within-cultivar variation contributed to the majority genetic variation (81.99%) among the six annual ryegrass cultivars in this study. By using multi-bulk strategy based on different filtering thresholds, the results suggested that bands frequencies of 40% could be used as a reliable standard for cultivar identification in annual ryegrass. Under this threshold, 12 SSR primer pairs (00-04A, 02-06G, 02-08C, 03-05A, 04-05B, 10-09E, 12-01A, 13-02H, 13-12D, 14-06F, 15-01C and 17-10D) were detected for direct identification of six tetraploid annual ryegrass cultivars used in this study, which could be incorporated into conservation schemes to protect the intellectual property of breeders, ensure purity for consumers, as well as guarantee effective use of cultivars in the future.

##  Supplemental Information

10.7717/peerj.7742/supp-1 Supplemental Information 1 The information of the SSR primers used in this study

10.7717/peerj.7742/supp-2 Supplemental Information 2 Genetic similarity coefficient data genetic similarity coefficient data

## Additional Information and Declarations

### Data Availability

The following information was supplied regarding data availability: The raw measurements are available in the Supplemental Files.
