# Supplementary material for: Genetic variability evaluation and cultivar identification of tetraploid annual ryegrass using SSR markers
Source: PeerJ. 2019 Sep 20;7:e7742. doi: 10.7717/peerj.7742 (PMC6756138; doi:10.7717/peerj.7742)
Supplement: Supplemental Information 1 [file peerj-07-7742-s001.docx]

| **SSR name** | **SSR motif** | **Forward primer** | **Reverse primer** |
| --- | --- | --- | --- |
| LMgSSR00-04A | (CA)16 | TATGTGGGCTAAGCCCCACG | CTTTGGCGGGAACTCTACCG |
| LMgSSR01-01E | (AC)18 | CGCAGCATCGTGGTCACTAGTT | GGCTGTGAGCCTGCCACTAGTAG |
| LMgSSR01-02H | (CA)31 | CAGTTGCAAAGCCGATTTCG | ACAGTTGGAGTTAACCCCATAGTCA |
| LMgSSR01-06D | (AC)14 | CACGTTCAGCCGGCTAGAGA | AAGATCGCTACGACCTGCGC |
| LMgSSR01-09C | (AC)3(GC)6(AC)11GC(AC)6 | ACAAAGCATACCTACAAAACGCACG | TCACTCAATCACAATCCCTCATGTG |
| LMgSSR01-10G | (AC)24 | CGCGTTCCTGCATCATTCAC | ACGAAGCCAACAACCGGCTA |
| LMgSSR02-05G | (AC)23 | GCAGTGGCTCCAGTGGCTTT | ACGGCTGGGAATCCACACTC |
| LMgSSR02-06G | (AC)34 | CTTTCAGCCGGACGACCAAC | TATAACGGCGGACACGACGA |
| LMgSSR02-07D | (AC)16 | GCCAGGACTATGGCCATGGA | GTGGACCGTTGTTGCCTTCC |
| LMgSSR02-08C | (CA)22 | CAACAACAGCAGCAGTTGCA | ACCCAGCACTGCTCCCAGTA |
| LMgSSR03-04E | (AC)16 | CTCAAAATGAGCCCGCCTTG | CCGACGATGATGACGCATTC |
| LMgSSR03-05A | (AC)15(GGA)14 | CCCTGGCTAGGGTTGGATCA | CACGCCCACACAGTCTCTCA |
| LMgSSR04-05B | (AC)33 | CGCACGTCAGTTCTCCCGTT | CGCGCCATCTGTACATCCCA |
| LMgSSR04-09D | (AC)20 | GCAAGCAATTGCATGCAG | TCTGCAGGGAGCACTGTTT |
| LMgSSR07-01D | (AC)34 | CTAGACCACACCGAGATGACCG | AAGGCCACTCAATCAAAGAGATTG |
| LMgSSR07-07G | (AC)15 | CAACCAGAGCACGCCCTACA | GCACATTGCCTTCCGTGATG |
| LMgSSR09-09C | (CA)12 | GCGTGCAACATGGAGACACC | CGGGCTTATAGATGTGCCCG |
| LMgSSR09-10H | (AC)14 | ACACACAACATGTAGAATCAAACCG | AGGTGGACATGACCGCTTCG |
| LMgSSR10-09E | (AC)12 | TCCAAGTGAACGAGTTGCGC | TCATCGTCACCACAGTGGCC |
| LMgSSR12-01A | (AC)22(TC)21(AC)17 | AAGCAGCACAAACAATGTGC | CTTTAGAACCTGCTGACGGC |
| LMgSSR13-02H | (TC)17(AC)10(GC)4(AC)8 | TGGCCTAGGGGACAGGTTCA | TGTATAGAGGAGCAANAATATGGATC |
| LMgSSR13-07A | (AC)14 | CACGGAGGCATTTGATTCCC | CGCGACCAGTTCCTCGATCT |
| LMgSSR13-12D | (AC)15 | TTGCTGCTGCACCAATAGCG | GAGCCGATGATGCCACATTC |
| LMgSSR14-06F | (CA)13 | GCAACGCCGTCATGACAGAA | TCCCTGCATTGTTCAAGCCA |
| LMgSSR15-01C | (AAC)26 | CCGCTGGGCAGCTAGTCATT | GATGCTTTCACTGCCACCGA |
| LMgSSR16-01E | (AC)9 | CGTTGTCTTGAGTGAATTGACTGC | CCTCTCTAACCACGTCGACTCG |
| LMgSSR16-06G | (AC)12 | CACATGGCATGCACAAACCA | GACGTGCCGACATGCAAAAC |
| LMgSSR17-04E | (AC)10 | CGAACGCCTACATGCATGCG | GAGCAGTTCGCCAGGGAGAA |
| LMgSSR17-10D | (CA)26 | CTTTCGCTCTAGCTTGATGTCTCC | AATCATTATGGAACATCGTGATTAGTG |

**Table S1 The information of the SSR primers used in this study**
